# Screening Enzymes That Can Depolymerize Commercial Biodegradable Polymers: Heterologous Expression of *Fusarium solani* Cutinase in *Escherichia coli*

**DOI:** 10.3390/microorganisms11020328

**Published:** 2023-01-28

**Authors:** Fernando Santos-Beneit, Le Min Chen, Sergio Bordel, Raquel Frutos de la Flor, Octavio García-Depraect, Raquel Lebrero, Sara Rodriguez-Vega, Raúl Muñoz, Rosa Aragão Börner, Tim Börner

**Affiliations:** 1 Institute of Sustainable Processes, Dr. Mergelina s/n, 47011 Valladolid, Spain; 2 Department of Chemical Engineering and Environmental Technology, School of Industrial Engineering, University of Valladolid, Dr. Mergelina s/n, 47011 Valladolid, Spain; 3 Nestlé Research, Société des Produits Nestlé S.A, Route du Jorat 57, 1000 Lausanne, Switzerland

**Keywords:** biodegradable polymer, depolymerization, cutinase, esterase, monomer, plastic

## Abstract

In recent years, a number of microbial enzymes capable of degrading plastics have been identified. Biocatalytic depolymerization mediated by enzymes has emerged as a potentially more efficient and environmentally friendly alternative to the currently employed methods for plastic treatment and recycling. However, the functional and systematic study of depolymerase enzymes with respect to the degradation of a series of plastic polymers in a single work has not been widely addressed at present. In this study, the ability of a set of enzymes (esterase, arylesterase and cutinase) to degrade commercial biodegradable polymers (PBS, PBAT, PHB, PHBH, PHBV, PCL, PLA and PLA/PCL) and the effect of pre-treatment methods on their degradation rate was assessed. The degradation products were identified and quantified by HPLC and LC-HRMS analysis. Out of the three enzymes, *Fusarium solani* cutinase (FsCut) showed the highest activity on grinded PBAT, PBS and PCL after 7 days of incubation. FsCut was engineered and heterologous expressed in *Escherichia coli*, which conferred the bacterium the capability of degrading solid discs of PBAT and to grow in PBS as the sole carbon source of the medium.

## 1. Introduction

Plastics are a type of polymeric material that are widely used in daily life. Due to their cheap production costs, high durability and strength, plastics are an excellent material to use for a variety of applications, such as the automotive industry and the packaging sector. Plastics have provided great societal benefits in terms of health and safety in food packaging [1]. However, plastic pollution has become a major global problem due to its constantly increasing production. Especially, single-use conventional petroleum derived plastics, i.e., polypropylene (PP) and polyethylene terephthalate (PET), show a high resistance to chemical and biological degradation [1]. As a result, between 1–2 million tons of plastics are estimated to enter the oceans annually, harming marine life and potentially also human health [2]. Bioplastics, a term comprising plastics that are bio-based (i.e., plastics synthesized from renewable sources), biodegradable or both, have been proposed as a promising alternative to petroleum derived plastics [3]. Some bio-based plastics, such as polyethylene (PE) and Nylon11, are non-biodegradable. Others, such as polyhydroxyalkanoates (PHAs), are both bio-based and biodegradable and are synthetized by certain bacteria in less than 30 min [4]. Although bioplastics only represent nowadays 1% of the total plastic production annually, the production capacity of biodegradable plastics is estimated to increase in the next decades [5]. These plastics, besides being more environmentally friendly, can be degraded into useful monomers and oligomers by microorganisms and enzymes, which might provide a new direction for a circular economy [6]. Developing efficient biotechnologies capable of transforming bioplastic waste into high value chemical building blocks or into the constituents of the original polymer offers promising routes towards life-cycle-engineered products. Research progress of relevant mechanisms with the latest biotechnological recycling strategies, including the use of different pre-treatments for (bio)plastic waste, microbial-based processes, key factors and mechanisms governing microbial degradation, can be found in [3].

Industries of different sectors are currently transitioning to biodegradable and bio-based packaging [7]. Ultimately, the advancement of new bio-based materials for packaging comes hand-in-hand with the development of recycling and degradation processes. Biodegradation is a biochemical process where microorganisms convert a material into harmless end-products, such as water, CO_2_ and new microorganisms. However, this process of biodegradation is not only highly dependent on the environmental conditions (e.g., marine environment, composting or landfilling), but also on the polymer structure and the microbiota itself. There are several circular end-of-life options suitable for biodegradable polymers: mechanical recycling, organic recycling and chemical recycling [3]. Mechanical recycling has been most widely used to recycle conventional and bio-based PE and PET. Organic recycling includes industrial composting and anaerobic digestion. During anaerobic digestion, the biodegradable material is converted into biogas, which can be used to produce renewable energy [8]. In industrial composting, the aerobic biodegradation process is performed in a controlled manner to produce compost, CO_2_ and water. However, composting from common biodegradable packaging materials (e.g., PHAs) would not add nitrogen and phosphorus to the soil since the composition of these plastics is formed by C, O and H [9]. Chemical recycling enables the recovery of the polymer building blocks (i.e., monomers), which can be reused to produce virgin polymers [5]. Chemical recycling provides a great circular end-of-life option [10], but more research needs to be performed to improve the cost-effectiveness of this process. In this sense, an emerging technology is enzymatic recycling, where the high specificity of enzymes is used to selectively degrade the polymer substrate into oligomers and monomers under mild reaction conditions with limited formation of by-products. Over the past decades, there has been an increasing number of studies published on the enzymatic degradation of biodegradable plastics [11,12,13]. However, due to the inconsistency between the studies, comparisons among them are quite challenging. Some studies lack the data on the properties of the polymer (e.g., crystallinity and melting point, T_m_), while others lack certain characteristics of the enzyme (e.g., optimal temperature, pH). Therefore, a better understanding of the degradation efficiency is necessary to assess the potential of enzymatic recycling as an end-of-life option for biodegradable polymers. Since the degradation efficiency is interconnected between the polymer properties, enzyme characteristics and the medium conditions, analysis of the enzymes and polymers separately are required.

The enzymatic degradation of polymeric materials is a surface erosion process (heterogeneous process), where the enzyme first adsorbs onto the solid polymer surface and then hydrolyses the polymer chains via its active site [14]. Therefore, not only the characteristics of the enzyme and the reaction conditions should be taken into account, but also the chemical and the physical properties of the polymer [11,12,13]. Chemical properties of the polymer such as the hydrophobicity and the molecular weight determine the rates of biodegradability. A higher molecular weight decreases the rate of polymer degradation, while a high hydrophobicity of the polymer may prevent an effective adsorption of the enzyme [15]. Additionally, the physical properties of the polymer, such as crystallinity, glass transition temperature (Tg), greatly affect the enzyme degradation capacity [11]. Overall, a polymer contains a crystalline and an amorphous part. The amorphous region contains more loosely packed molecules, making this region more prone to enzymatic attack. By milling the polymer into fine particles, a higher surface-to-volume ratio can be achieved, which in turn may lead to a higher enzyme accessibility and degradation rates [15,16]. Therefore, the effect of milling as pre-treatment method on the physical-chemical properties of the polymer resins should be investigated.

Lipases, cutinases, esterases, PHA depolymerases, proteases and catalases have been reported to have polyester degrading activities [12]. In this study, we have selected three enzymes from the literature (esterase, arylesterase and cutinase) that are reported to have a huge potential for bioplastic degradation [12,13]. Esterases are hydrolasing enzymes that catalyse the breakdown and formation of ester bonds. Aromatic compounds are the natural substrates for arylesterases, having a high potential to degrade aliphatic polyesters, such as PBAT. Cutinases hydrolyse cutin, a plant polymer that covers the aerial surfaces of a plant. Cutin is an insoluble polyester of C16 and C18 hydroxy fatty acids. As cutinases lack the hydrophobic lid covering the active side, it allows the enzyme to hydrolyze a great variety of substrates [17,18]. In particular, in this study we have selected a cutinase from the phytopathogenic fungi *Fusarium solani* (FsCut cutinase) (E.C. 3.1.1.74), an esterase from *Alcanivorax borkumesis* (AbEst esterease) (EC. 3.1.1.1) and an arylesterase from *Pseudomonas pseudoalcaligenes* (PsEst arylesterase) (E.C. 3.1.1.2). Wallace et al. [16] reported high degradation rates of PBAT with the *P. pseudoalcaligenes* PsEst arylesterase enzyme, but PsEst did not degrade PLA or PET [19]. On the other hand, AbEst showed hydrolytic activity on PDLA, PCL and PHBV on agarose plate assays [20]. Hu et al. [21] reported the highest *F. solani* FsCut activity for emulsions of poly (butylene succinate-co-adipate) (PBSA), followed by emulsions of PBS and PCL. Similar results were obtained by Murphy et al. [22]. Finally, Zumstein et al. [23] also reported activity of the enzyme against PBAT.

The aim of this work is to determine the hydrolysis activity of these proteins towards commercial biodegradable polyesters and to understand which parameters are necessary for improving their degradation efficiency. The study also aims at selecting the enzyme with the best degradation profile and to express it in the most studied bacterial model (i.e., *Escherichia coli*).

## 2. Materials and Methods

### 2.1. Chemicals, Enzymes and Polymers

All materials and chemicals used were purchased from Sigma-Aldrich or VWR unless stated otherwise. The *Fusarium solani* cutinase, *Alcanicorax borkumensis* esterease and *Pseudomonas pseudoalcaligenes* arylesterase recombinant enzymes were provided commercially by Evoxx (Monheim am Rhein, Germany). Biodegradable polymers were purchased from the following commercial trade names: PHB (ENMAT™ Y3000P), PHBH (Danimer, Bainbridge, GA, USA), PHBV (ENMAT™ Y1000P, approx. 3% valeric acid content), PCL-440744 (Sigma, St. Louis, MO, USA), Blend PLLA/PCL 80/20 (PLLA Luminy^®^ L105/PCL Capa 6500D, ITENE, Valencia, Spain), PLA (Luminy^®^ L105), PBAT (M·VERA^®^ B5037, Bio-Fed, Köln, Germany) and PBS (BioPBS™ FZ91PM/FZ91PB from PTT MCC Biochem, Bangkok, Thailand). The plastic materials, which were initially in a pellet form, were grinded in a commercial blender (Cecotec Titanium 2000 pro, Valencia, Spain) equipped with titanium blades. Repeated crushing (~3 min on, ~5 min off) using dry ice as a cooling strategy was employed to avoid melting and recrystallization, as reported elsewhere [6]. Finally, the polymer powders were sieved using an electromagnetic sieve (CISA RP-20, Barcelona, Spain) with stainless-steel sieves of 100, 250, 500 and 1000 μm and then dried at room temperature. The different powder fractions were stored in closed packaging under dark and dry conditions at room temperature until usage. Prior to the experiments the resins and powders were washed once with 0.1% Sodium Dodecyl Sulfate (SDS) solution, three times with Milli-Q water, once with 70% ethanol and then 100% ethanol. The resins and powders were subsequently dried in a SpeedVac concentrator (Savant, Barnstable, MA, USA) until complete dryness.

### 2.2. Characterization of the Crystallinity of the Polymer by Differential Scanning Colorimetry

The thermal properties of the polymers were analysed using differential scanning colorimetry (DSC) using a TA Instruments DSC 25 (New Castle, DE, USA). Approximately 9–11 mg of resin or polymer powder sealed in Tzero Pans with Tzero lid with pin hole (TA instruments, USA) were subjected to a heating-cooling-heating cycle at a rate of 10 °C/min. The measurement for the polymers PCL, PBAT, PBS and PHBH was performed from 0 to 180 °C, starting for 5 min at 0 °C, heating up to 180 °C and kept for 5 min for the first run, cooling down to 0 °C and kept for 5 min and heated up to 180 °C for the second heating run. For the polymers with a higher Tm, PLLA, PHB, PHBV and PLLA/PCL blend, the measurement was conducted from 0 to 220 °C.

To determine the degree of crystallinity of the sample, the following formula was used: *Xc* = Δ*Hm*0 × 100% [24]. *Xc* is the degree of crystallinity. Δ*Hm* is the enthalpy during the melting. Δ*Hm*0 is the enthalpy for the normalized enthalpy values (J/g) for 100% crystalline polymer, as reported in the literature: for PHB = 146 J/g [25], PLLA = 93.1 J/g [26], PBAT = 114 J/g [27], PBS = 110.3 J/g [28], PCL = 139.5 J/g [29] and PHBH = 146 J/g [29]. PHBV was assumed to have the same enthalpy as PHB [30].

### 2.3. Esterase Activity Assay with Para-Nitrophenol Butyrate

Para-nitrophenol butyrate (pNPB) was used as a substrate to determine the esterase activity of the enzymes. A stock solution of 10 mM pNPB in 2-propanol was prepared and stored at −20 °C. Enzyme solutions were prepared freshly by dissolving the lyophilizate crude extracts in Milli-Q water. Solutions of 0.8 mM pNPB were prepared freshly in buffer (0.1 M K_2_HPO_4_/KH_2_PO_4_). The pNPB substrate solution was warmed up at 37 °C for 5 min prior to the measurement. The enzyme solution (10 μL), diluted to a suitable concentration, and pNPB solution (190 μL) were added in a NUNC 96-well plate (Thermo Fisher Scientific, Waltham, MA, USA). For the blank measurement, Milli-Q water was added instead of the enzyme to follow, if any, the auto-hydrolysis of pNPB. The release of para-nitrophenol (pNP) by hydrolysis was followed for 30 min at 37 °C by measuring the absorbance at 405 nm using a plate reader (Varioskan^®^ Flash microplate reader, Thermo Fisher Scientific, USA). The concentration of released pNP was calculated based on the standards (0.01–0.8 mM) for all the pH tested (6.5, 7, 7.5 and 8). The initial rates (activity) for pNP product release were determined through linear regression of the initial product formation (Supplementary Figure S1). The measurements were performed in duplicate. The protein content was determined by the Bradford assay.

### 2.4. Thermal Stability of FsCut

The thermal stability of FsCut was analyzed by incubating the enzyme at different temperatures and then determining the residual activity in a separate assay using pNPB as substrate. Enzyme stock solutions were divided into five aliquots and subjected to heat treatment for 0, 15, 30, 60 and 120 min at five different temperatures (60, 55, 50, 45 and 37 °C.) After the heat treatment, the enzyme stock solutions were briefly centrifuged to separate the aggregated insoluble from the soluble protein fraction and to retrieve the condensation collected in the lid of the sample tube. One unit was defined as the amount of enzyme required to hydrolyze the conversion of 1 μmol of pNPB per minute under the specified assay conditions.

### 2.5. Ultrasonic Treatment of Polybutylene Adipate Terephthalate (PBAT)

To create the polymer melt, 0.5 g of polybutylene adipate terephthalate (PBAT) resins were dissolved in 10 mL chloroform (volumetric flasks) using gentle stirring for 2 h. In a 30 mL Pyrex^®^ tube, 1 mL of the polymer melt with 10 mL ice cold buffer (0.1 M K_2_HPO_4_/KH_2_PO_4_, pH 7.5) was sonicated for 5 min pulsating (4 s on/2 s off, 24 Watts, 45% amplitude using a Digital sonifier 450^®^, Branson, MO, USA) in an ice bath. The dispersion was transferred into a 25 mL beaker and the remaining chloroform was evaporated for 1 h under a gentle nitrogen stream (0.2 bar) while stirring at 300 rpm to obtain a dispersion of 0.5% *w*/*v*.

### 2.6. Enzymatic Hydrolysis of Biodegradable Polymers

In a 4 mL or a 2 mL glass vial, 9–11 mg of polymer powder (<1000 μM) or 900 μL of dispersion (containing ca. 4.5 mg polymer) was added. The 4 mL vials were closed with PTFE/silicone/PTFE septum and the 2 mL vials with a polypropylene cap featuring a PTFE/silicone septum. An aliquot of 900 μL of buffer (0.1 M K_2_HPO_4_/KH_2_PO_4_, pH 7.5) and 100 μL of (15 min, 37 °C pre-warmed) enzyme solution was added to obtain a final total protein concentration of 0.25 (2 mL vials) or 1 mg/mL (4 mL vials). Buffer was added instead of enzyme to the polymer powder as a blank. The vials containing the polymer powder were incubated at 37 °C in an ISF-X incubator shaker (Kuhner AG, Birsfelden, Switzerland) at 100 rpm in a horizontal position to keep the powder suspended. The vials were incubated for 7 days for both the 0.25 mg/mL and 1 mg/mL protein content. The vials containing dispersed PBAT were incubated at 37 °C in a thermoshaker (Bioshake IQ, QInstruments, Jena, Germany) at 500 rpm for 7 days. Samples of 50 μL were frequently taken for the product analysis by HPLC. The vials containing dispersed PBAT were briefly centrifuged (2 min) before sampling the supernatant. As positive control, a base hydrolysis was performed with 2 M NaOH. An aliquot of 1 mL 2 M NaOH was added to 9–11 mg polymer powder and incubated at 50 °C at 1200 rpm overnight in a ThermoMixer^®^ 5437 (Eppendorf, Hamburg, Germany). Samples of 50 μL were taken for the product analysis by HPLC.

### 2.7. Analysis of Degradation Products by High-Performance Liquid Chromatography (HPLC)

The degradation products of the biodegradable polymers were identified by high-performance liquid chromatography (HPLC). Samples of 50 μL were transferred to ice and acidified with 50 μL 0.6% phosphoric acid in order to halt the reaction. For the samples containing 2 M NaOH, 100 μL 1 M HCl was added to neutralize the sample. The samples were then immediately centrifuged at 16,000× *g* at 0 °C for 15 min and 50 μL of the supernatant was transferred to an HPLC vial containing an insert. A 5-μL aliquot of the sample was injected into the HPLC (Agilent 1200 series) equipped with a reverse-phase C18 HPLC column (Synergi™ 4 μm Hydro-RP 80 Å, 150 4.6 mm, Phenomenex, Torrance, CA, USA) and a DAD-detector. The mobile phases were 0.1% phosphoric acid in MQ-water (A) and 0.1% phosphoric acid in acetonitrile (B). 100% acetonitrile (C) and 100% MQ-water (D) were used for cleaning. To separate all the products, a 15-min elution gradient was used at a flowrate of 1 mL/min. The column temperature was maintained at 40 °C and the sample rack at 10 °C. The compounds were detected at 210 nm and 241 nm with a reference wavelength at 550 nm and a reference bandwidth at 100 nm by the DAD. The UV-spectrum was recorded ranging from 190–400 nm with a 2 nm spectrum step. Stock solutions of the degradation products were prepared in 10 mM; lactic acid (LA), succinic acid (SA), 3-hydroxybutyric acid (3HB), 6-hydroxyhexanoic acid (6HH) and adipic acid (AA) were dissolved in MQ-water. Terephthalic acid (TPA) was dissolved in DMSO. Calibration standards were prepared from these stock solutions.

### 2.8. Identification of Degradation Products by Liquid-Chromatography High Resolution-Mass Spectrometry (LC-HRMS)

The identification was performed via an UHPLC system (TLX-2) with Allegro quaternary pumps coupled to an Orbitrap Q-Exactive mass spectrometer (Thermo Fisher Scientific, San José, CA, USA) and to a diode array detector (DAD, Ultimate 300RS, Thermo Fisher Scientific, Milan, Italy) placed in series with a corona CAD (Corona Veo RS, Thermo Fisher Scientific, San José, CA, USA) for data acquisition. The mass spectrometer was fitted with a heated ESI source (HESI, Thermo Fisher Scientific, San José, CA, USA) and the split between the mass detector and the DAD and corona CAD detectors in series was 1:9. A volume of 5 μL was injected on a reversed phase Acquity BEH C8 analytical column (100 mm × 2.1 mm × 1.7 μm) (Waters Corporation, Milford, MA, USA) kept at 40 °C. The flow rate was set at 0.4 mL/min. Both mobile phases were composed of 0.5 mM ammonium acetate and 0.1% formic acid in water (A) and methanol (B). The LC gradient used for the separation of the compounds is shown in Supplementary Figure S2.

The positive and negative ionization switching mode was operated with parameters as follows: sheath gas flow 15 arbitrary units (AU); auxiliary gas flow of 5 AU; sweep gas flow of 1 AU; capillary temperature of 250 °C; heater temperature of 100 °C; spray voltage of +3500 kV and −2500 kV for the positive and negative modes, respectively; S-lens radio frequency of 70 AU. Positive and negative HRMS data were acquired simultaneously in full scan (FS) and variable data independent acquisition (vDIA) mode. Resolving power full width half minimum (FWHM) were used at 35.000 @200 and 17.500 @200 for FS and vDIA mode, respectively. Acquisition was operated in FS mode over *m*/*z* range of (80–1200) and vDIA mode over five isolation mass windows in the quadrupole: (95–205), (195–305), (295–405), (395–505) and (495–1005). The normalized collision energy (NCE) was ramped between 20% and 60% for vDIA mode. Automatic gain control (AGC Target) was set at the dynamic range 1 × 106 and maximum injection time (IT) at 100 ms. DAD chromatograms were obtained with an analytical wavelength set at 254 nm and a bandwidth at 5 nm. CAD parameters were as follows: CAD evaporator temperature (EVT) of 35 °C, data collection rate of 10 Hz, a noise filter of 3.6 sec and a general power function value (PFV) of 1.

### 2.9. Construction and Cloning of a Synthetic Cutinase Gene in Escherichia coli

A custom synthetic cutinase gene (with *E. coli* optimized codons) was ordered for “de novo” synthesis by GeneScript. The synthetic gene was designed to be controlled by the promoter of the *E. coli gapA* gene (coding for a glyceraldehyde-3-phosphate dehydrogenase). The coding protein was designed to include the *E. coli* TorT leader sequence (MRVLLFLLLSLFMLPAFS) in substitution to the native signal peptide (MKFFALTTFLAATASA), which was predicted by both PrediSi and SignalP-5.0 servers. The synthetic construction was cloned upstream from a transcriptional terminator harbored in vector pEX-1 (OriGene Technologies, Inc., Rockville, MD, USA). The constructed vector encoding the synthetic cutinase gene, and the pEX-1 vector without the synthetic gene, were transformed into *E. coli* One Shot™ BL21 Star™ (DE3) resulting in E11 and EX1 strains, respectively.

### 2.10. PBS and PBAT Degradation Activity Assays

E11 and EX1 strains were grown in glass bottles with LB Broth (Miller, L3522 Sigma-Aldrich) or MSM [31] media at 250 rpm and 37 °C. After growing the strains for 16 h, the cells were pelleted by centrifugation and the supernatants were filtered using sterile Millipore disposable 0.22 µm syringe filters. Filtered supernatants were mixed in sterile bottles with 3 g of grinded plastic (sterilized by UV radiation, i.e., to avoid re-polymerization of the polymer by autoclaving) or with the same amount of plastic previously melted and sterilized inside a glass bottle by autoclaving (i.e., formation of a sterile plastic disc is produced after cooling down). The bottles containing the filtered supernatants and plastic materials were hermetically closed with an isoprene rubber and an aluminum crimp seal and incubated at 37 °C during several days depending on the experiment.

PBAT and PBS degradation was estimated by quantification of 1,4-butanediol release (a monomer constituting both polymers) in the reaction samples. 1,4-butanediol determination was processed by collecting 2 mL-samples with a sterile needle, following by a centrifugation step (14,000 rpm for 5 min at 4 °C) and the filtering of the supernatant using Nylon syringe filters of 0.22 µm. Filtered samples were analyzed using an Agilent 7820A GC coupled with a 5977E MSD (Agilent technologies, Santa Clara, CA, USA) equipped with a DB-wax column (30 m × 250 μm × 0.25 μm). The detector and injector temperatures were kept constant at 250 °C and the oven temperature was increased from 50 °C to 220 °C at 10 °C min^−1^ and maintained at 220 °C for 2 min, before being increased again at 5 °C min^−1^ until reaching 240 °C. Instrument linearity was evaluated with 1,4-butanediol (Reagentplus 99%, Sigma-Aldrich) in the concentration range of 0–64 mM by integration of the area of a single peak acquired at ~14.09 min (Supplementary Figure S3).

## 3. Results and Discussion

### 3.1. Selection of Enzymes with Polyester Degrading Activity and Characterization of the Crystallinity of Pre-Treated Polymer Powders

A selection of three enzymes (esterase, arylesterase and cutinase) was made based on the literature [11,12,13]: (a) cutinase from the phytopathogenic fungi *Fusarium solani* (FsCut cutinase) (E.C. 3.1.1.74), (b) esterase from *Alcanivorax borkumesis* (AbEst esterease) (EC. 3.1.1.1), (c) arylesterase from *Pseudomonas pseudoalcaligenes* (PsEst arylesterase) (E.C. 3.1.1.2). Table 1 shows an overview of the three enzymes and their reported capability to degrade a range of polymers.

The main biodegradable polymers tested in this work (including information related to synthesis, structure and applications) are shown in Supplementary Table S1. The thermal properties of these polymers were analysed using DSC (see an example in Supplementary Figure S4) and the results obtained are shown in Supplementary Table S2. Information about the crystallization and melting parameters for the different biodegradable polymers of the study might help to develop pre-treatment methods for enhancing enzymatic depolymerization. For example, decreasing the crystallinity of the polymer would be a great option to improve degradation efficiency as it is reported in [33]. The formation of crystallinity is directly correlated with the melting temperature [34]. Grinding polymer resins can lead to high temperatures and a local melting of the polymer. Once the melted polymer slowly cools down again, it can arrange itself into crystalline structures, hence increasing the crystallinity of the polymer [35].

In summary, the crystallinity of the polymer powder was in general slightly lower than that of the resin. For almost all the polymers, also a slight decrease in the melting temperature was observed. Only for PHBV a slight increase of 2.8% was noted. The decrease in crystallinity could be explained by the grinding method [35,36]. In this work, the polymers were ground together with dry ice. During the grinding, some parts of the polymer may have exhibited high temperatures, causing an increase in amorphous parts of the resin. This would have been immediately cooled by the presence of dry ice, preventing any crystallite formation in the polymer and keeping the amorphous disorder of the melt. In conclusion, the pre-treatment method followed in this work caused little changes to the ratio of amorphous and crystalline fraction (compare Supplementary Table S2) and is thus not significantly influencing the outcome of the enzymatic degradation of polymers.

### 3.2. Effect of pH on the Esterase Activity of the Enzymes

To estimate the esterase activity of the enzymes on long fatty acid model substrates, a high-throughput method using para-nitrophenol butyrate (pNPB) as substrate [37] was employed. Insoluble model substrates such as para-nitrophenol palmitate (pNPP) can cause turbidity in the substrate solution, as it is not miscible with water [37]. Besides, the turbidity can also affect the absorbance of the measurement. In the literature, the addition of gum arabic, sodium deoxycholate and Triton X-100 (2%) emulsifiers are widespread used to estimate the esterase activity of lipases and esterases [37,38]. Using a *Candida* sp. lipase, we showed that addition of Triton X-100, gum arabic and sodium deoxycholate resulted in a loss of 60–70% activity of the lipase on pNPB (Supplementary Figure S5). Therefore, we excluded the use of these emulsifiers for assessing the activity of the enzymes towards long chain fatty acids. Similar results have been reported in the literature, where addition of Triton X-100 completely inhibited the activity of diverse lipases [39].

**Figure 1:**
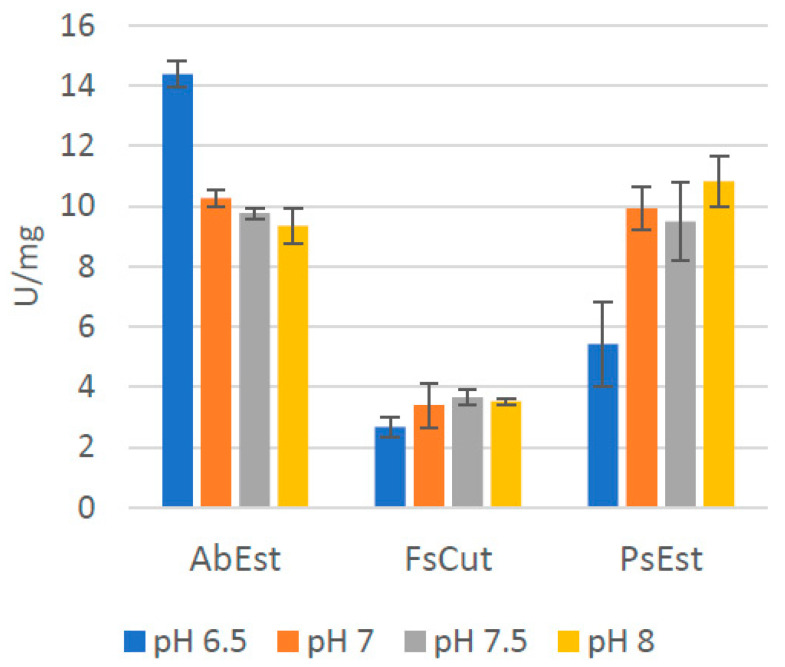
Effect of pH on the esterase activity (expressed in units per mg of protein) using 0.8 mM para-nitrophenol butyrate (pNPB) at 37 °C and different pH solutions. One unit was defined as the amount of enzyme required to hydrolyze the conversion of 1 μmol of pNPB per minute under the specified assay conditions. The plots represent an average of the reaction in duplicate with their respective deviation as error bars.

To determine the effect of pH on enzyme activity the pNPB assay was used (as described in Material and Methods) and the release of para-nitrophenol (pNP) was measured (see Figure 1). The esterase activity of AbEst (14.4 ± 0.43 U/mg) and PsEst (10.8 ± 0.83 U/mg) were found to be the highest at pH 6.5 and pH 8, respectively, followed by FsCut (3.6 ± 0.26 U/mg) at pH 7.5. Hajighasemi et al. [20] have reported an optimal pH of AbEst between pH 9–11, unlike pH 6.5 observed in this study. This difference is likely due to the use of a different model substrate (α-naphthyl propionate) in their study. For FsCut, pH 8 was reported to support its optimal activity for PBS degradation [21], which is similar to the findings in this study with pNPB. Likewise, Wallace et al. [16] reported am optimum pH for PsEst between pH 7–8 for pNPB, matching also with the results of this study.

### 3.3. Enzymatic Degradation of Biodegradable Polymer Powders

The degree of degradation of the biodegradable polymers by the enzymes was tested at a protein concentration of 0.25 and 1.0 mg/mL with the ground polymer powders (particle size < 1000 µm). The ability of the enzyme to degrade the polymer was determined by HPLC analysis of the degradation products (see Material and Methods section).

Polybutylene adipate terephthalate (PBAT):

PBAT is a co-aromatic-aliphatic polyester consisting of terephthalic acid (TPA), adipic acid (AA) and 1,4-butenediol (BD) units. Depending on the enzyme cleavage site, different degradation products are possible. Supplementary Figure S6A shows the different monomers and oligomers that were identified by LC-HRMS, and which were assigned to the corresponding retention times of the HPLC method.

**Figure 2:**
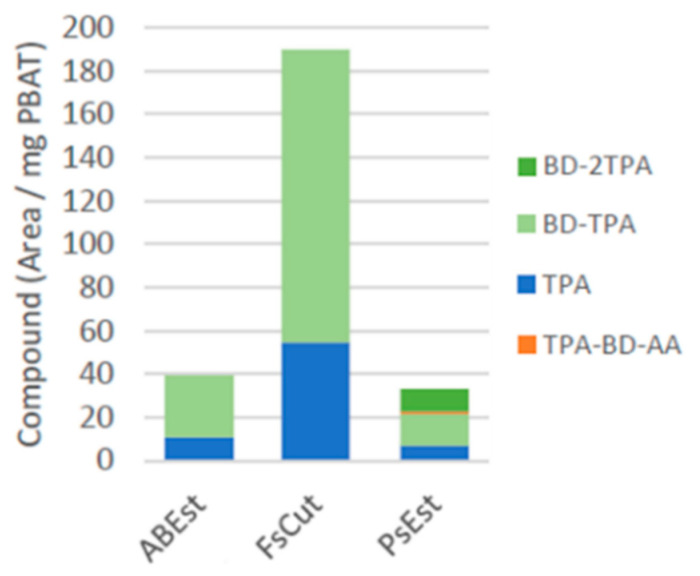
Degradation products of PBAT with the corresponding enzymes at a protein concentration of 1 mg/mL after 6.9 days of incubation (normalized per mg PBAT and subtracted blank).

For PBAT, the highest activity was detected for FsCut, followed by AbEst, with the main degradation products being TPA and BD-TPA after 7 days for both enzymes (Figure 2). To our knowledge there are no literature reports of AbEst directly degrading PBAT. The highest TPA content after 6.9 days was observed for FsCut (0.518 and 0.126 mM, for 1 and 0.25 mg/mL protein content, respectively). TPA concentrations of 0.076 and 0.0322 mM were determined for AbEst and PsEst (at a protein content of 1 mg/mL), respectively. Any concentrations lower than 0.01 mM could not be accurately quantified due to the calibration range but were detected for AbEst and PsEst at 0.25 mg/mL protein content.

For FsCut, large concentrations of the dimer BD-TPA were also identified. Lower concentrations of this dimer were observed for AbEst and PsEst. Low concentrations of TPA-BD-AA and BD-2TPA were detected for PsEst, whereas no trimers were identified for FsCut or AbEst over the course of the entire experiment. This suggests that both enzymes preferably cleaved between the TPA-BD ester bond and the BD-AA (position 1 and 2; Supplementary Figure S6B).

Poly-butylene succinate (PBS):

Hydrolysis of PBS can occur at two different ester bonds (Supplementary Figure S6C). Both the dimer succinic acid-butanediol (SA-BD) and the monomer succinic acid (SA) were detected by HPLC. FsCut was the only enzyme that showed hydrolysis of PBS. The quantified concentration of succinic acid (SA) at the end of the reaction was 0.92 mM and >1.2 mM at 0.25 and 1 mg/mL protein content, respectively. The ability of FsCut to degrade PBS has been described elsewhere [21,32]. However, the quantification of SA after enzymatic hydrolysis has never been reported.

Poly-caprolactone (PCL):

PCL consists of only one monomeric compound, 6-hydroxy hexanoic acid (6HH). Enzymatic hydrolysis with FsCut showed large concentrations of 6HH after incubation and no other peaks of 6HH oligomers were observed (Supplementary Figure S6D). The concentrations obtained for 6HH with both 0.25 and 1 mg/mL protein contents were larger than 12 mM. No 6HH was detected for AbEst or PsEst. AbEst has been reported to degrade PCL (see Table 1) but, unfortunately, we do not have an explanation for the lack of degradation observed in this study. It is reported that FsCut is able to gradually decrease the crystallinity of a PCL film. However, compared to *Candida antarctica* lipase, FsCut is considered a less good candidate for PCL degradation [40]. Another report in the literature identified that pH 9–10 improves the PCL hydrolysis activity by FsCut [22]. No reports were found directly stating the quantity of the degradation products.

Poly-lactic acid (PLA) and PLA/PCL copolymer:

No degradation was detected for the PLA containing polymers for all enzymes tested (data not shown). The lack of degradation could be explained by the high glass transition temperature (Tg) of ~60 °C [11] and Tm of ~175 °C of the polymer (Supplementary Table S2). As the reaction was performed at 37 °C, the polymer chains of PLA were possibly not flexible enough for enzymatic hydrolysis. The same applies for the PLA/PCL copolymer. No hydrolysis products were detected for FsCut, whereas, it was highly active on the degradation of PCL, suggesting that a high PLA content (80%) may prevent the chain flexibility required for hydrolysis by FsCut. The results correspond to what is reported in the literature (none of the enzymes have been reported to hydrolase PLA). AbEst was found to be active against poly(D,L-lactide) [20] but not against poly(L-lactide), which is the polymer used in this study.

Polyhydroxy alkanoates (PHA):

Three commercial PHAs, namely poly(3-hydroxybutyrate) (PHB), poly(3-hydroxybutyrate-co-hydroxyvalerate) (PHBV) and poly(3-hydroxybutyrate-co-3-hydroxyhexanoate) (PHBH) were used to assess the ability of the enzyme for PHA degradation. The main degradation products of these polymers are 3-hydroxybutyric acid (3HB), 3-hydroxyvaleric acid (3HV) and hydroxyhexanoic acid (3HH). Besides monomers, dimers and trimers are also common products of enzymatic hydrolysis of PHAs [41]. No degradation products of 3HB, 3HV and 3HH or its dimers or trimers were detected after the enzymatic treatment of the PHAs with the tested enzymes (data not shown). Both FsCut and AbEst are reported to degrade PHAs (see Table 1). Low chain motility could be a reason why no hydrolysis products were detected for the PHAs in this study, since the reaction was performed at 37 °C and the Tm for the PHAs are relatively high (see Supplementary Table S2).

In summary, FsCut showed a high activity on grinded PBAT, PBS and PCL after 7 days releasing the monomers TPA, SA and 6HH at 0.518 mM, >1.25 mM and >12.06 mM, respectively (i.e., and with degrading efficiencies of: >0.63%, >1.26% and >13.4%, respectively). Hu et al. [21] reported highest FsCut activity for emulsions of PBSA, followed by emulsions of PBS, PCL and PLLA. AbEst and PsEst hydrolyzed grinded PBAT into TPA after 7 days, releasing 0.076 mM and 0.033 mM of the monomer, respectively. No activity was detected for any of the enzymes for the rest of polymers (see a summary of the results in Table 2).

### 3.4. Effect of Particle Size on the Degradation Rate of PBAT

The goal of using grinded polymer pellets was to investigate the intrinsic degradability of the polymer by enzymes without the effect of processing. As particle size [6] and processing including pre-treatment can alter the molecular structure and morphology, this work aimed at studying the effect of particle size using grinding as a tool to create different size fractions but not changing the degree of “historical” crystallinity found in the original pellet. As discussed above and shown in Supplementary Table S2, due to the insignificant change in amorphous and crystalline content the enzyme-catalyzed degradation rate should mainly depend on the particle size, enzyme activity and specificity as well as reaction conditions.

**Figure 3:**
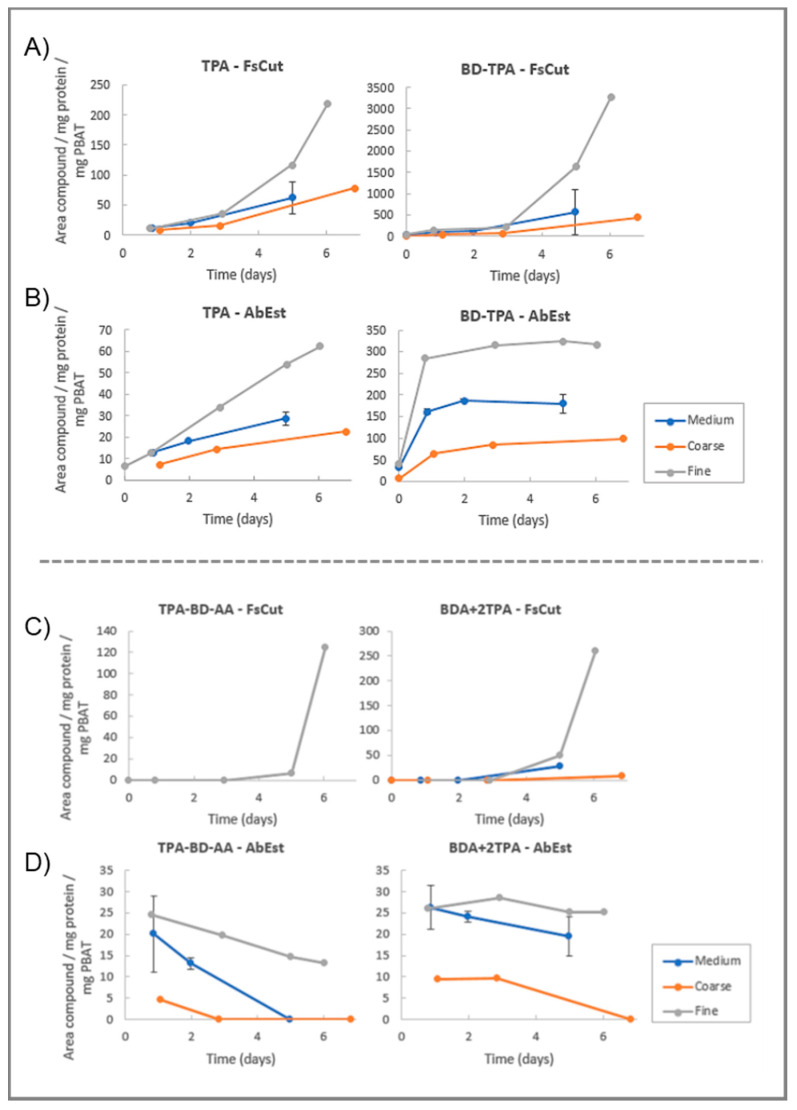
Enzymatic hydrolysis of different particle sizes of PBAT by FsCut (**A**,**C**) and AbEst (**B**,**D**) at 0.25 mg/mL protein content for each case. Particle sizes of PBAT are: coarse (orange), medium (blue) and fine (grey). The data points are corrected for the exact amount of protein content and PBAT, as well as a blank subtraction. For the medium particle size, the points represent an average of the reaction in duplicate with their respective deviation as error bars. Note that for the fine and coarse sizes no replicates were included in the study.

As FsCut and AbEst showed the highest hydrolytic activity toward PBAT, these enzyme-polymer combinations were further studied to investigate the effect of the polymer size on the degradation efficiency. In Figure 3 is shown the enzymatic hydrolysis assays for FsCut and AbEst (at 0.25 mg/mL protein content) with different particle sizes of PBAT: fine (<100 µm), medium (100–500 µm) and coarse (500–1000 µm).

For FsCut, the observed TPA content at end of the incubation period was increased by more than 2-fold when the finest dispersion was used compared to the coarse or medium powder (Figure 3A). A similar trend in the release of TPA was observed for AbEst but with a more linear trend in comparison to FsCut (Figure 3B). Interestingly, FsCut and AbEst exhibited completely distinct trends for BD-TPA. FsCut showed a lag-phase of 2–3 days, after which the hydrolysis rate increased, while AbEst exhibited a saturation-type trend (Figure 3A,B). With both enzymes, the TPA and BD-TPA release was higher with the smallest particle size, indicating that particle size is an important factor for the degradation rate. These distinct trends suggest that AbEst favors hydrolysis of PBAT at position 2, between BD and AA (Supplementary Figure S6B) and remains active for 6 days for the hydrolysis of BD-TPA to TPA.

For FsCut, no trimers (BD-2TPA and TPA-BD-AA) were identified over the course of the entire experiment for medium and coarse dispersions, in agreement with previous results. With the fine dispersion, low amounts of BD-2TPA and TPA-BD-AA were detected after 6 days incubation (Figure 3C). This shows that a large particle size prevents certain activity of the enzyme, while reducing the particle size allows for a better accessibility of FsCut to release trimers (and thus increases the degree of degradation). For AbEst, small amounts of trimers were detected throughout the experiment, mainly with fine and medium sizes (Figure 3D). This suggests that both enzymes preferably cleave between the TPA-BD ester bond and the BD-AA (position 1 and 2; Supplementary Figure S6B).

**Figure 4:**
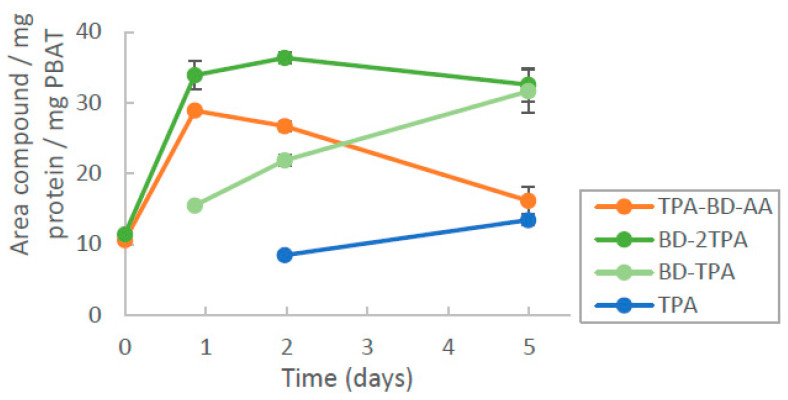
Enzymatic hydrolysis of medium particle size PBAT by PsEst over 5 days. The points represent an average of the reaction in duplicate with their respective deviation as error bars.

The change over the four analyzed hydrolysis products of PBAT (TPA, BD-TPA, BD-2TPA and TPA-BD-AA) was also tested for PsEst, but only with medium size PBAT (Figure 4).

A sharp increase in the products BD-2TPA and TPA-BD-AA was detected initially, followed by a gradual increase in BD-TPA and TPA and a decrease in BD-2TPA and TPA-BD-AA. This indicates that PsEst preferentially first hydrolyses the bonds between AA-BD (position 2 and 3; Supplementary Figure S6B) and then converts the produced trimers to the dimer and monomer TPA.

### 3.5. Thermal Stability of FsCut

**Figure 5:**
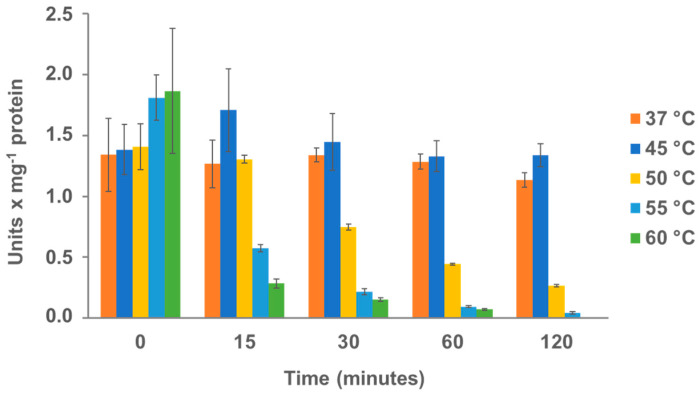
Thermal stability of FsCut. The thermal stability of FsCut was analyzed by incubating the enzyme at different temperatures and then determining the residual activity in a separate assay using pNPB as substrate at their corresponding temperatures. Enzyme stock solutions were divided into five aliquots and subjected to heat treatment for 0, 15, 30, 60 and 120 min at five different temperatures (37, 45, 50, 55 and 60 °C). The esterase activity (pNPB) is expressed in units per mg protein. One unit was defined as the amount of enzyme required to hydrolyze the conversion of 1 μmol of pNPB per minute under the specified assay conditions. The plots represent an average of the reaction in duplicate with their respective deviation as error bars.

The hydrolysis rate of polymers is highly dependent on the temperature, i.e., higher temperatures results in more movement of the polymer chains, increasing enzyme adsorption and thus promoting hydrolysis [9]. However, since enzymes may lose their activity at higher temperatures, it is essential to assess the enzymes on their thermostability to define their limitations. The previous results have shown that FsCut displays the highest activity in a range of polymers, including PBAT, PBS and PCL (Table 2). Therefore, we decided to check the thermal stability of this enzyme for further applications. To this aim, the esterase activity of FsCut was analyzed using pNPB as a substrate after incubation of the enzyme at different temperatures ranging from 37 to 60 °C (see Figure 5).

FsCut showed maximum activities at 55 and 60 °C with time 0 incubation. However, a great loss of activity was observed after the initial 15 min of incubation at 55 and 60 °C, which is likely due to the unfolding of the enzyme. At 50 °C, a 50% decrease was observed after 30 min of incubation (but not in the first 15 min of incubation, as it was the case of the incubations at highest temperatures). This is in agreement with previous work in which the activity of FsCut, produced by a recombinant *Saccharomyces cerevisiae* strain, was maintained in the first 10 min of reaction at 50 °C [42]. Up to 90% of activity was also retained after 10 min of incubation (but with a much higher temperature, i.e., 85 °C) of FsCut from a recombinant *E. coli* strain [43]. FsCut maintained around 100% of its activity after incubation at 37 and 45 °C for two hours (being the magnitude of both activities similar). This is also similar to the results reported for FsCut, cloned and overexpressed in *Pichia pastoris*, in which the enzyme showed 75% relative activity on PBS degradation after 1-h incubation at 40 °C [21]. It is likely that the small difference (100% vs. 75%) observed between both studies is due to the different reaction conditions employed.

In conclusion, the enzyme activity was higher with higher temperatures, but the protein was quite sensible to incubation at temperatures above 45 °C.

### 3.6. Construction and Checking of a Synthetic FsCut Gene for Optimal Expression in E. coli

As we have shown before, FsCut displayed the highest activity in a range of polymers and maintained more than 50% of its activity after incubation at 37 °C, which renders it in a good candidate to be heterologous expressed in a bacterial platform for in vivo studies. *F. solani* cutinase (FsCut) has already been expressed in a number of heterologous hosts [44,45,46,47,48]. However, we attempted to the construction of a new heterologous strain to check our findings in vivo and using the most employed bacterial host, *Escherichia coli*.

To degrade the plastic polymers, the heterologous expressed protein has to be secreted to the extracellular medium, since these polymers do not go through the cell membrane of the bacterium. To achieve this goal, the native sequence of the *F. solani* FsCut gene was substituted by a signal peptide (SP) recognized by the *E. coli* machinery. In particular, a SP from the Signal Recognition Particle (SRP) translocation pathway was selected (TorT). TorT SP has been shown to result in much higher translocation levels of a number of proteins in comparison to the conventional SPs recognized by the SEC or TAT translocation pathways [49]. To express the protein in *E. coli* efficiently, the *gapA* promoter was fused to the open reading frame of FsCut. The *E. coli gapA* promoter is a constitutive promoter that works efficiently under both aerobic and anaerobic conditions [50]. Finally, to avoid possible expression problems arising from the presence of a large percentage of low usage codons of the native cutinase gene in *E. coli*, the open reading frame of the gene was codon optimized for its use in the host bacterium (detailed information on the construction of the vector is described in the Material and Method section). The constructed FsCut synthetic gene was cloned into pEX-1 vector (Origene) resulting in the construct named pEX-FsCut. This vector was cloned into *E. coli* BL21 Star™ (DE3), which was further referred as to E11 strain. The vector without the cutinase (pEX-1) was cloned in the same *E. coli* bacterial system, resulting in the EX1 control strain.

**Figure 6:**
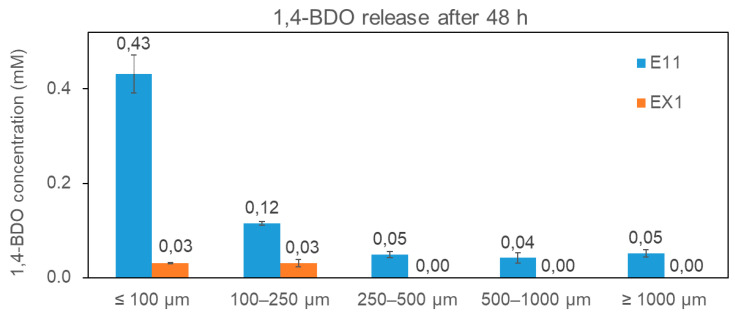
Enzymatic hydrolysis of different particle sizes of PBS by E11 and EX1 culture supernatants over 2 days at 37 °C (estimated by measurement of 1,4-butanediol release; 1,4-BDO). The plots, with their respective deviation as error bars, represent an average of each reaction in duplicate (the average value is also shown as a number on top of each bar).

To check if the E11 strain expresses and exports a functional FsCut enzyme to the culture medium, a PBS degradation assay was performed with the supernatants of both E11 and EX1 cultures. To this aim, LB cultures of E11 and EX1 strains were grown for 16 h and then, 25 mL of supernatant of each culture were filtered and mixed with 3 g of grinded PBS granules with different sizes (≤100 µm; 100–250 µm; 250–500 µm; 500–1000 µm; ≥1000 µm). Mixtures of supernatants and plastic granules were incubated at 37 °C for 48 h (with two technical replicates). Samples for 1,4-butanediol (1,4-BDO) determination (released after PBS degradation) were collected at time 0 (before the incubation) and time 48 h (after the incubation) and analyzed by GC-MSD as described in Material and Methods. As expected, at time 0, when no PBS degradation is possible, no 1,4-BDO was detected with any of the two supernatants (data not shown). After 48 h (see Figure 6), 1,4-BDO was released in the E11 samples with all particle sizes analyzed, which might indicate that the enzyme is functional (since the control strain, which does not contain FsCut, completely lacked activity with the largest PhBS particle sizes and retained just basal levels of 1,4-BDO release with the smallest granule sizes (≤100 µm and 100–250 µm).

Therefore, similar to the results observed before for PBAT, the size of the grinded polymer pellets affected significantly the PBS degradation activity of the enzyme.

### 3.7. E11 Strain Can Degrade PBAT Films in a Short Scale of Time

**Figure 7:**
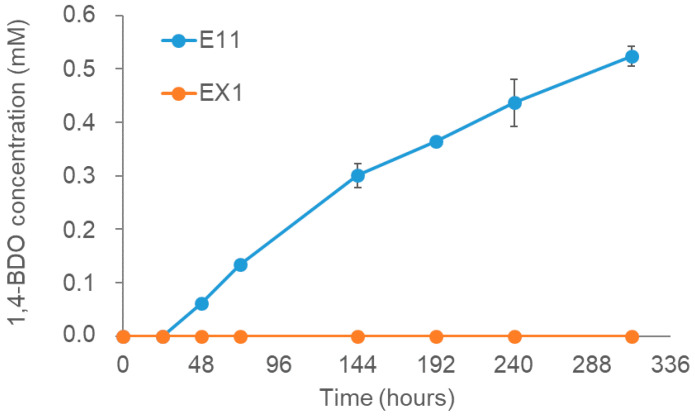
Enzymatic hydrolysis of PBAT blocks by E11 and EX1 culture supernatants over 2 weeks at 37 °C (estimated by measurement of 1,4-butanediol release; BDO). Vertical error bars correspond to the standard error of the mean of two replicated experiments.

Finally, the ability of E11 enzymatic broth to degrade PBAT and PBS discs (instead of grinded polymer) was investigated. The experiment was conducted by mixing 50 mL of filtered supernatants of either E11 or EX1 cultures with a 3-g plastic disc (of either PBAT or PBS) located at the bottom of a 120 mL glass bottle (see Supplementary Figure S7). The glass bottles were hermetically closed with an isoprene rubber and an aluminum crimp seal and incubated at 37 °C for 2 weeks. Samples for 1,4-BDO quantification (which is released after degradation of both PBS and PBAT polymers) were taken periodically during the experiment and analyzed by GC-MSD as described in Material and Methods. The results shown in Figure 7 indicated a positive degradation activity of FsCut over the PBAT discs after 48 h of incubation and afterwards. The activity was maintained constantly at least for 2 weeks, in contrast to the control strain that did not release any 1,4-BDO during the entire duration of the experiment, which indicates that PBAT degradation activity is due to the heterologous expressed FsCut synthetic gene and no because an indirect capability of the strain. Curiously, no degradation of PBS discs was observed for any of the strains during the time of the experiment (data not shown).

In vivo degradation of PBS (a plastic that is typically used for film packaging) by the strain would be of most interest for sustainable microbial recycling strategies. PBS is produced by the direct esterification of succinic acid (SA) with 1,4-BDO, therefore, and as we have shown before, after degradation of this polymer, SA and 1,4- BDO monomers are released to the culture medium. *E. coli* cannot metabolize 1,4- BDO (as we have shown in the study), but it can directly catabolize SA as carbon and energy source throughout the Krebs cycle. Therefore, the engineered E11 strain should be able to grow on PBS as the sole carbon source in the culture medium after proper degradation of the polymer in its monomers. Preliminary studies have shown that E11 can grow on cultures with grinded PBS material as the sole carbon source (contrary to the control strain that cannot). However, under these conditions, growth rates are yet very low (see Supplementary Figure S8); possibly due to limitations in the degradation yields achieved by the enzyme under the conditions of the experiment. Nevertheless, this result is a good starting point for developing further studies on plastic degradation by this strain, which might include valorization of plastic residues on valuable compounds, such as enzymes or metabolites.

## 4. Conclusions

The objective of this work was the screening of enzymes that can degrade commercial biodegradable polymers and to introduce the best candidate into a model bacterial strain. To this aim, the degradation capacity of a set of enzymes with esterase, arylesterase and cutinase activities over a series of commercial biodegradable polymers was evaluated. The effect of pre-treatment methods on the degradation rate of the polymers was also evaluated via DSC. In general, the pre-treatment method of grinding the polymer powder with dry ice (followed in this work) slightly decreased the crystallinity for all polymers, except for PHBV. The enzymatic hydrolysis of the ground polymers was performed over 7 days at 37 °C with protein concentrations of 0.25 and 1.0 mg/mL. The degradation products were identified and quantified by HPLC and LC-HRMS analysis. FsCut (cutinase) was the enzyme that degraded the higher number of substrates and with the highest activity on PBAT, followed by AbEst. Enzymatic hydrolysis by FsCut in fine dispersed PBAT compared to the coarse dispersion increased the TPA release by more than 2-fold and revealed the release of trimers not observed with the larger particle sizes. Degradation profiles of AbEst (esterase) and PsEst (arylesterase) on PBAT showed that the bottleneck for both enzymes is the release of the TPA monomer. A potential way to accelerate complete hydrolysis of PBAT could be done by combining FsCut with either AbEst or PsEst, as PsEst and AbEst exhibited high activity for the release of trimers and FsCut for the release of TPA and the dimer BD-TPA. *E. coli* in vivo studies with an engineered secreted FsCut enzyme showed that PBS degradation (in addition to PBAT) is significantly affected by the particle size of the grinded plastic polymer. Interestingly, the engineered FsCut enzyme was able to degrade PBAT solid discs, but not those made with PBS. The developed *E. coli* strain can grow on grinded PBS as sole carbon source of the culture media, and it could provide a sustainable platform for the valorization of plastic residues via microbial fermentation.

## Figures and Tables

**Table 1:** Enzymes used in this study and their reported polymer degradation capability.

Name	Organism	Enzyme Type	PDB	Reported Activity on Polymers	References
AbEst	*Alcanivorax borkumensis*	Esterase	-	PLA, PCL, PHBV, PBS, PHB	[20]
PsEst	*Pseudomonas pseudoalcligenes*	Arylesterase	4JGG	PBAT	[16,17,18,19]
FsCut	*Fusarium solani*	Cutinase	1AGY	PBSA, PBAT, PBS, PCL, PLA, PHB	[21,22,23,32]

**Table 2:** Summary of the degradation potential of the enzymes on the bioplastic polymers by HPLC analysis based on the released monomers after 7 days incubation at 37 °C with protein concentrations of 0.25 and 1.0 mg mL^−1^, respectively. ^a^ pNPB activity (U/mg) at pH 7.5, ^b^ Protein content (mg/mL). Values obtained that fall outside of the calibration range were shown with either (>) or (<). No detected activity is indicated with “ND”. Please, note that the table does not include the results with the polymers (PHB, PHBH, PHBV, PLA and PLA/PCL) since no activity was detected for any of them with any of the enzymes.

		PBAT TPA (mM)		PBS SA (mM)		PCL 6HH (mM)	
Name	pNPB Activity ^a^	0.25 ^b^	1 ^b^	0.25 ^b^	1 ^b^	0.25 ^b^	1 ^b^
AbEst	9.78 ± 0.18	<0.01	0.076	ND	ND	ND	ND
PsEst	9.50 ± 1.30	<0.01	0.033	ND	ND	ND	ND
FsCut	3.65 ± 0.26	0.126	0.518	0.92	>1.25	>12.0	>12.0

## Data Availability

The authors declare that all data obtained have been included into the manuscript, its additional files and/or repositories.

## Supplementary Materials

The following supporting information can be downloaded at: https://www.mdpi.com/article/10.3390/microorganisms11020328/s1.
