# Supplementary material for: Screening Enzymes That Can Depolymerize Commercial Biodegradable Polymers: Heterologous Expression of Fusarium solani Cutinase in Escherichia coli
Source: Microorganisms. 2023 Jan 28;11(2):328. doi: 10.3390/microorganisms11020328 (PMC9963400; doi:10.3390/microorganisms11020328)
Supplement: Supplementary file 1 [file microorganisms-11-00328-s001.zip › microorganisms-2149134-supplementary.pdf]

Supplementary material

Screening enzymes that can depolymerize commercial biodegradable polymers: heterologous expression of *Fusarium solani* cutinase in *Escherichia coli*

Fernando Santos-Beneit[1,2,*], Le Min Chen[3,†], Sergio Bordel[1,2], Raquel Frutos de la Flor[2], Octavio García-Depraect, Raquel Lebrero, Sara Rodriguez-Vega[1,2], Raúl Muñoz[1,2], Rosa Aragão Börner[3], Tim Börner[3,‡,*]

1. Institute of Sustainable Processes, Dr. Mergelina s/n, 47011 Valladolid, Spain

2. Department of Chemical Engineering and Environmental Technology, School of Industrial Engineering, University of Valladolid, Dr. Mergelina, s/n, 47011 Valladolid, Spain

3. Nestlé Research, Société des Produits Nestlé S.A, Route du Jorat 57, 1000 Lausanne, Switzerland

* Corresponding author: fernando.santos.beneit@uva.es

† Current address: Department of Biotechnology, Delft University of Technology, Van der Maasweg 9, 2629 HZ Delft, Netherlands.

‡ Current address: HES-SO Valais/Wallis, School of Engineering, Institute of Life Technologies, Rue de l’Industrie 19, 1950 Sion, Switzerland

Supplementary Tables

Table S1

Polymer	Application examples	Source	Structure
Polylactic acid (PLA)	Films, containers, coatings for paper and cardboard	Lactic acid monomer from renewable resources	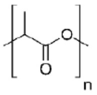
Polyhydroxy alkanoate (PHA)	Bottles, containers, sheets, films	Naturally produced by bacteria	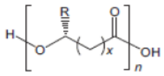
Polybutylene succinate (PBS)	Bottles, bags, disposable tableware	(bio-based) succinic acid and 1,4-butanediol	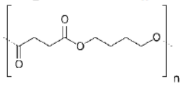
Polybutylene adipate terephthalate (PBAT)	Bags, cling wrap, coating	Synthesis of terephthalate, adipic acid and 1,4-butanediol	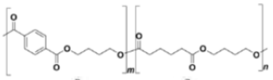
Polycaprolactone (PCL)	Trash bags	Synthesis via ring-opening polymerization of Ɛ-caprolactone	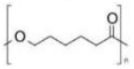

Table S1. Main biodegradable polymers classified as bio-based or fossil-based with applications examples, synthesis and structure.

Table S2

Polymer	Material	Tm_1 (°C)	ΔH_m (J/g)	X_c (%)
PCL	resin	63	75.6	54.17
	powder	61	66.9	47.98
PBAT	resin	122	12.5	10.98
	powder	119	10.4	9.16
PBS	resin	119	75.0	67.95
	powder	115	71.7	64.96
PHBH	resin	144	68.6	47.00
	powder	142	64.6	44.26
PLA	resin	178	54.7	58.74
	powder	175	51.2	55.02
PHB	resin	178	87.1	59.65
	powder	176	84.9	58.17
PHBV	resin	176	79.8	54.63
	powder	175	83.9	57.44
PLA/PCL	resin PLA	176	38.8	41.69
	resin PCL	66	18.3	13.14
	powder PLA	175	38.2	41.00
	powder PCL	61	19.7	14.13

Table S2. Crystallization and melting parameters for different biodegradable polymers in its resin and powder form (particle size < 100 μm). Melting temperature of the first heating (Tm1). Melting enthalpy ($\Delta \boldsymbol{Hm}$). Crystallinity ($\boldsymbol{Xc}$).

Supplementary Figures

Figure S1

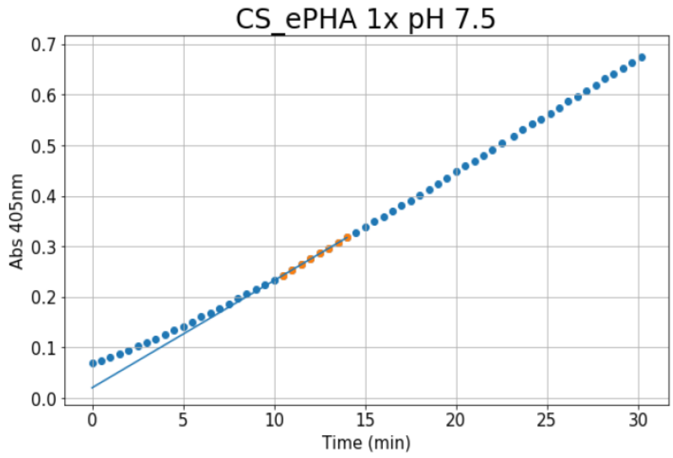

Fig. S1. A typical plot of the Python script of the change in absorbance at 405nm due to the release of pNP. The orange points indicate the position of which the activity was calculated from with the blue line as the regression line over those points.

Figure S2

	% solvent	
Time (min)	A	B
0	95	5
0.5	95	5
2.5	65	35
10	0	100
18	0	100
18.5	95	5
22	95	5

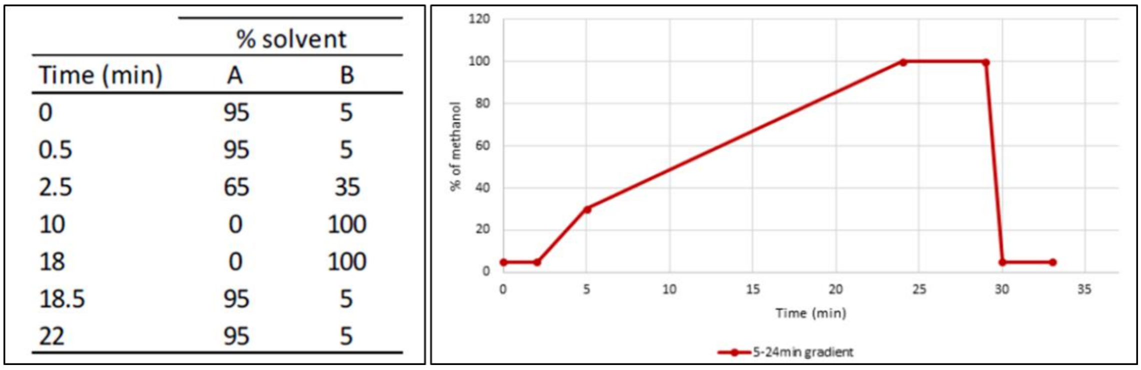

Fig. S2. Table and figure showing the LC chromatographic gradient coupled to HRMS used for separation of the compounds, using 0.5 mM ammonium acetate and 0.1% formic acid in water (A) and methanol (B).

Figure S3

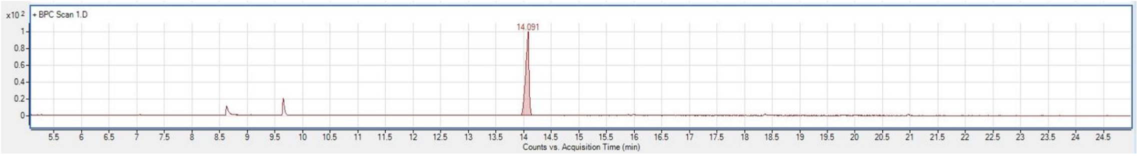

Fig. S3. GC-MSD analysis of 1,4-butanediol (99% purity) employ to detect PBS and PBAT degradation.

Figure S4

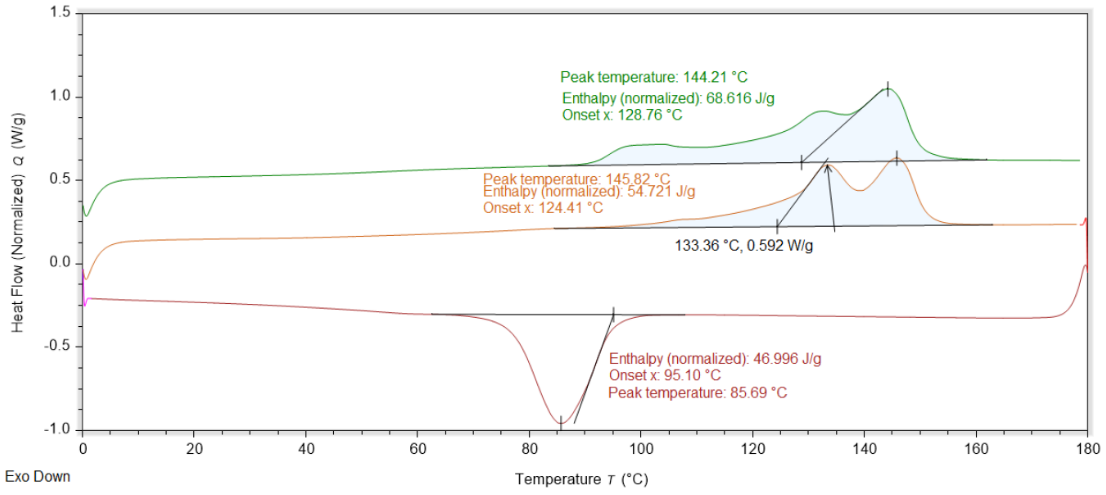

Fig. S4. DSC spectrum of PHBH resin. Green: First heating run, Red: first cooling run, Orange: second heating run. The curve of the first run of the DSC spectrum has a peak at around 100 °C. During the second run, this peak is not visible anymore, thus this peak was not part of the intrinsic values of the polymer. The first heating run provides information about the thermal history and post-processing of the polymer together with the intrinsic properties of the polymer, whereas the second heating run gives solely information about the intrinsic ("non-process") properties of the polymer.

Figure S5

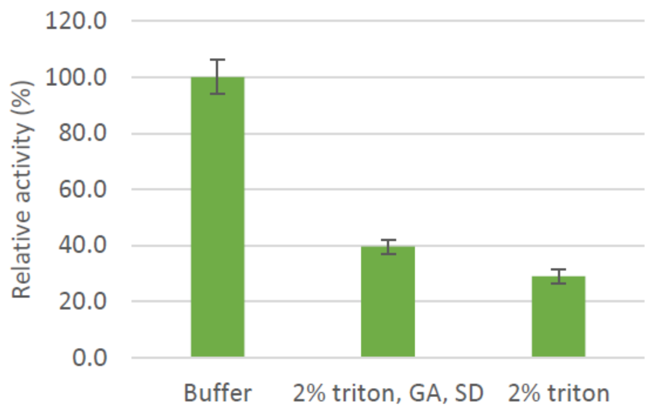

Fig. S5. The effect of triton, gum arabic (GA) and sodium deoxycolate (SD) on the esterase activity of pNPB of *Candida* sp lipase. The bars represent an average of the reaction in duplicate with their respective deviation as error bars. The substrate solution containing 200 µL Triton-X-100, 10 mg gum arabic, 20 mg sodium deoxycholate was prepared in buffer pH 7.5 (0.1 M K_2HPO_4/KH_2PO_4) with a final concentration of 0.8 mM pNPB. Alternatively, a second substrate solution with 200 µL Triton-X-100 was prepared to assess the effect of Triton-X- 100 on *Candida* sp. lipase.

Figure S6

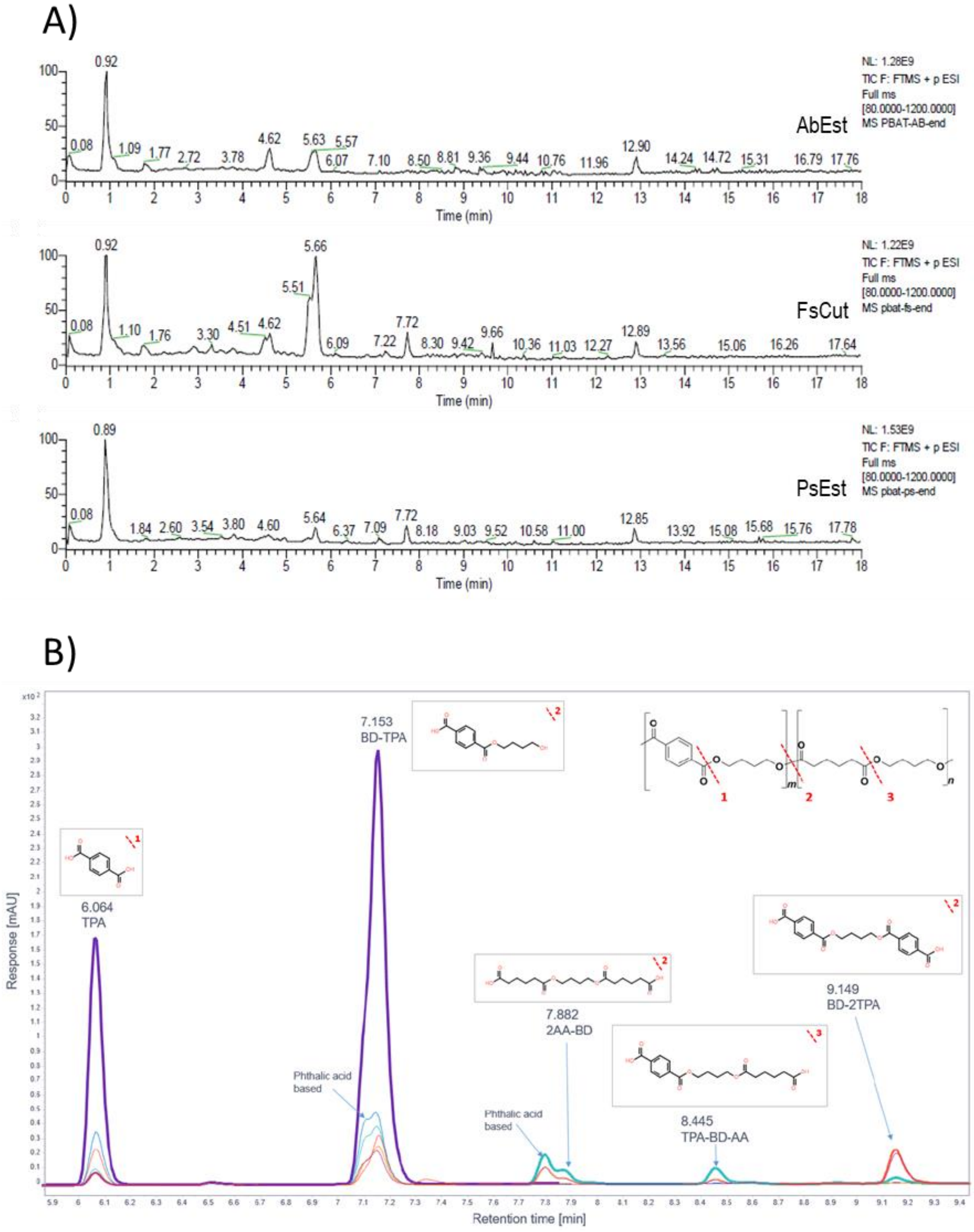
A)
NL: 1.28E9
TIC F: FTMS + p ESI
Full ms
[80.0000-1200.0000]
MS PBAT-AB-end
AbEst
NL: 1.22E9
TIC F: FTMS + p ESI
Full ms
[80.0000-1200.0000]
MS pbat-fs-end
FsCut
NL: 1.53E9
TIC F: FTMS + p ESI
Full ms
[80.0000-1200.0000]
MS pbat-ps-end
PsEst
Time (min)
B)
Response [mAU]
Retention time [min]
6.064
TPA
7.153
BD-TPA
Phthalic acid based
7.882
2AA-BD
8.445
TPA-BD-AA
9.149
BD-2TPA
1
2
3

C)

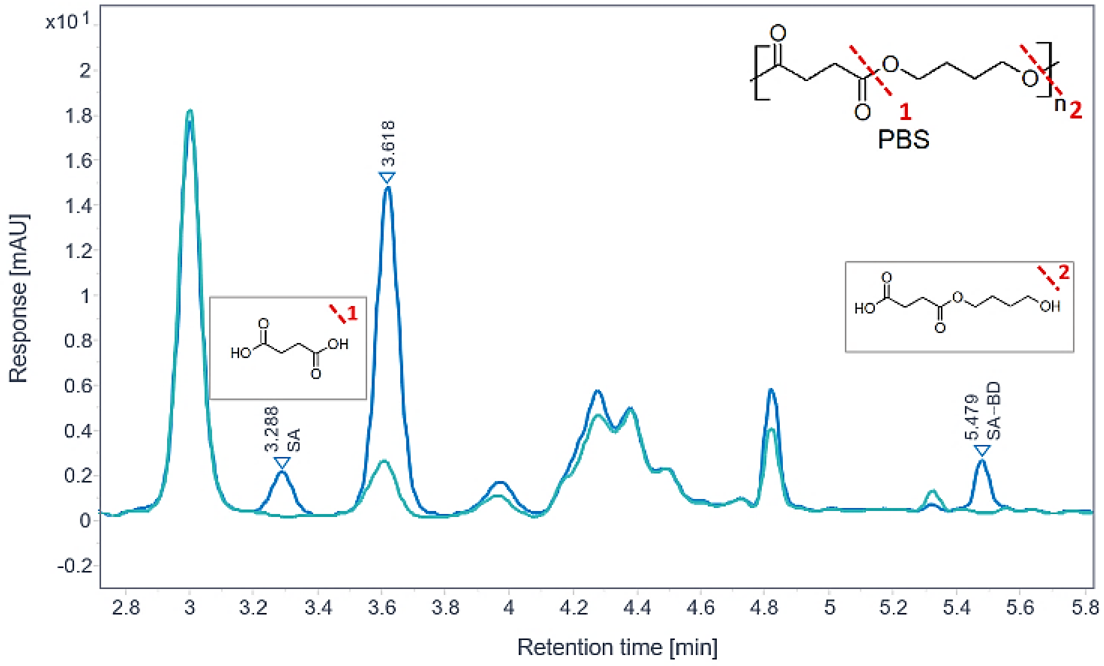

D)

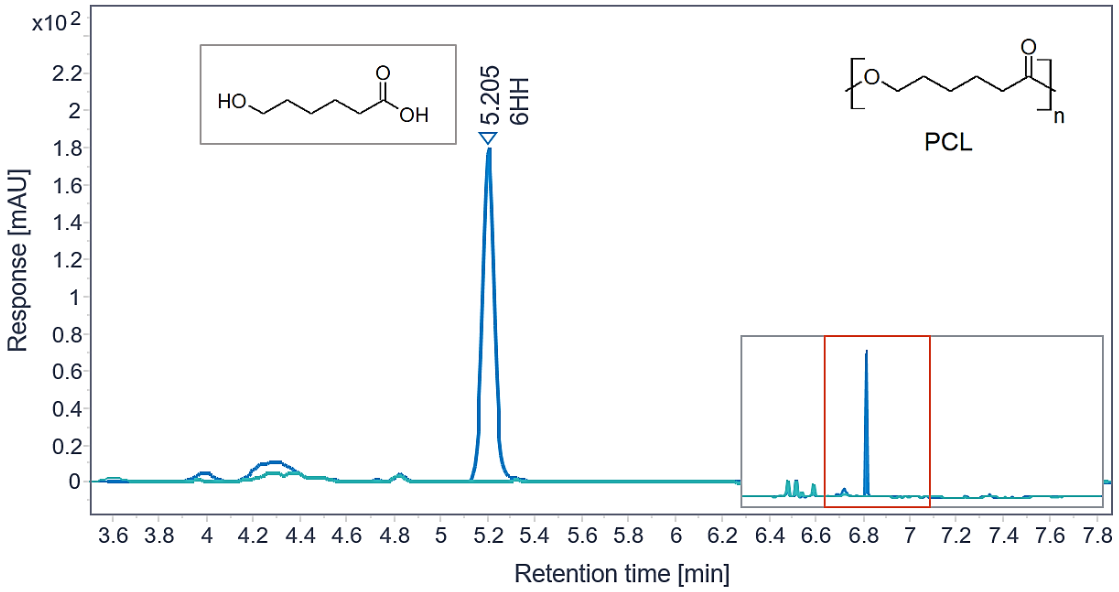

Fig. S6. Different monomers and oligomers identified by LC-HRMS. A) HRMS chromatograms from AbEst, FsCut and PsEst after 6.9 days with 1 mg/mL protein. The detected compounds were BD-TPA (5.66 min), BD-2AA (6.36 min), TPA-BD-AA (7.11 min) and the phthalic acid based compounds (5.51 min and 6.3 min). **B)** PBAT and the detected degradation products (after 6.9 days incubation, 1 mg/mL protein content) by AbEst (blue), FsCut (purple), PsEst (orange) and other enzymes not included in this work. The monomers detected by HPLC

were terephthalic acid (TPA), 1,4-butanediol (BD) and adipic acid (AA). Cleavage positions are indicated as a red dashed line. Cleavage at position 1 results in TPA, position 2 results in BD-TPA or 2AA-BD or BD-2TPA, position 3 in TPA-BD-AA. **C)** PBS and its hydrolysis products by FsCut (0.25 mg/mL protein) after 6.8 days incubation detected by HPLC consisting of succinic acid (SA; RT 3.289 min) and the dimer succinic acid-butanediol (SA-BD; RT 5.480 min). Cleavage positions are indicated as a red dashed line. Cleavage at position 1 results in SA, position 2 results in SA+BD. **D)** PCL and its hydrolysis product by FsCut (0.25 mg/mL protein) after 6.8 days incubation detected by HPLC consisting of 6-hydroxyhexanoic acid (6HH; RT 5.205 min). Blue: FsCut incubated with PCL, Green: Blank, FsCut incubated in only buffer. The bottom right chromatogram represents the entire chromatogram, framed in red is the current view.

Figure S7

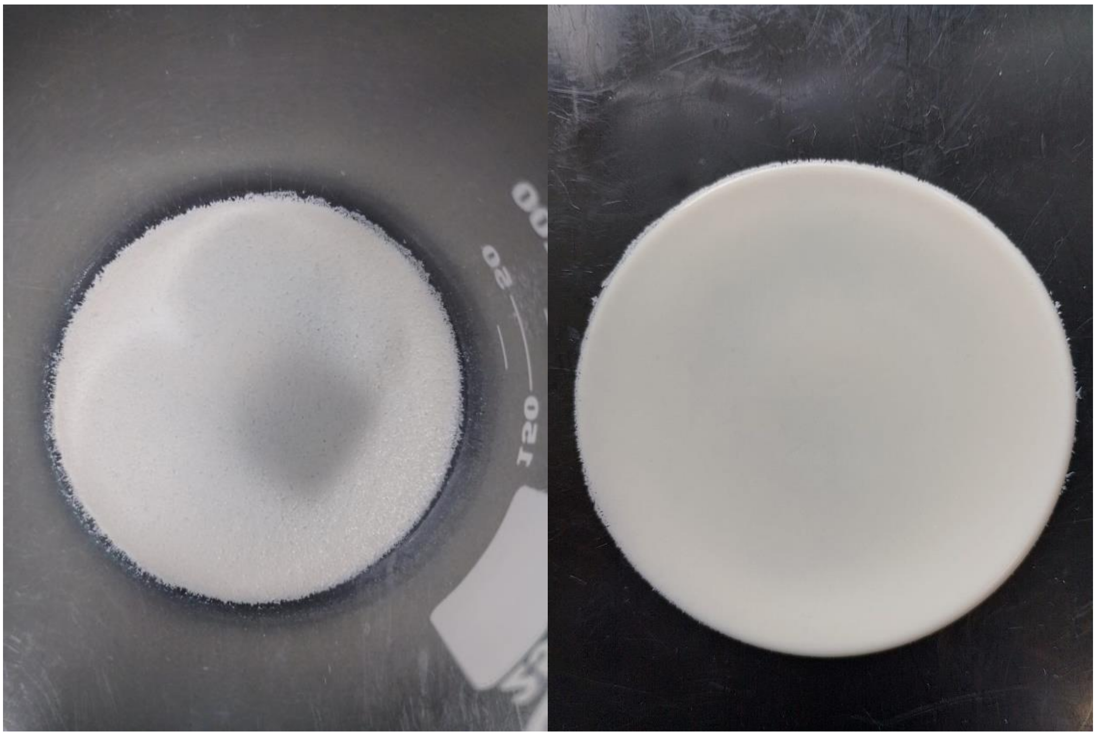

Fig. S7. PBS and PBAT discs used for the *E. coli* in vivo experiments. For each experiment 3 grams of plastic grinded polymer (100-250 µm) are introduced in glass bottles and melted by autoclaving (i.e. 121 ºC during 30 minutes). The formation of the sterile plastic disc is produced after cooling down. PBAT disc top view (left image), PBAT disc bottom view (right image).

Figure S8

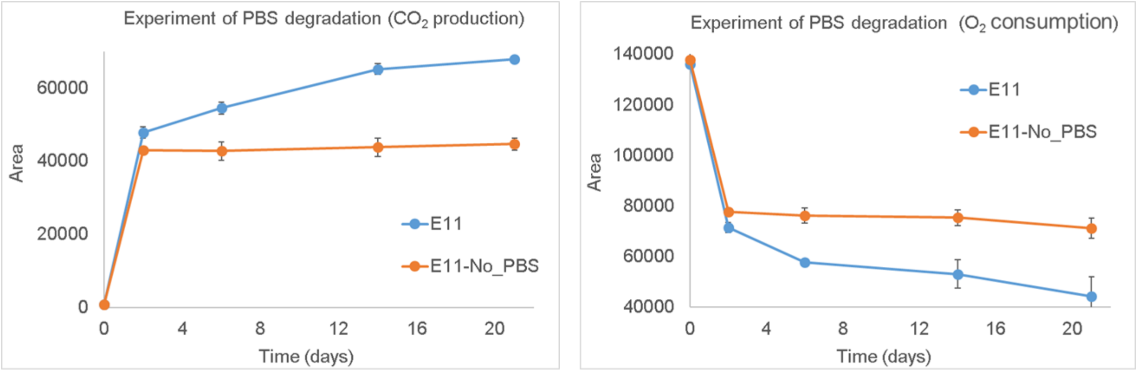

Fig. S8. *E. coli* E11 strain can degrade PBS and grow using the succinate released from the PBS degradation. A preculture of E11 strain in mineral salts medium (MSM) with succinate as sole carbon source (34 mM) was grown up to a OD_{600} of ~ 0.55 (i.e. exponential phase). A 1 mL sample of this preculture was used to inoculate 20 mL of fresh MSM media containing 1 gram of PBS grinded plastic (size of 100-250 µM) as sole carbon source or no PBS (control condition). Cultures were performed in hermetic closed bottles, which allows to measure CO_2 production and O_2 consumption (using a gas chromatographer) and to estimate the growth of the strain in each condition. Cultures were done in duplicate for each condition and run in parallel during 21 days at 37 ºC and 250 rpm. Vertical error bars correspond to the standard error of the mean of two replicated cultures.
